# MXene Quantum Dot/Zeolitic Imidazolate Framework Nanocarriers for Dual Stimulus Triggered Tumor Chemo-Phototherapy

**DOI:** 10.3390/ma15134543

**Published:** 2022-06-28

**Authors:** Xin Feng, Mingjun Li, Jianming Wang, Xianrui Zou, Hongshui Wang, Donghui Wang, Huan Zhou, Lei Yang, Wei Gao, Chunyong Liang

**Affiliations:** 1 Tianjin Key Laboratory of Materials Laminating Fabrication and Interface Control Technology, School of Materials Science and Engineering, Hebei University of Technology, Tianjin 300130, China; fengxin970204@163.com (X.F.); wjm28218987942021@163.com (J.W.); zouxianrui@163.com (X.Z.); 2 Center for Health Science and Engineering, School of Health Sciences and Biomedical Engineering, Hebei University of Technology, Tianjin 300130, China; limingjun0000@126.com (M.L.); kingflood@hebut.edu.cn (H.W.); wdh_81@163.com (D.W.); zhouhuan@hebut.edu.cn (H.Z.); ylei@hebut.edu.cn (L.Y.); 3 Department of Interventional Therapy, National Clinical Research Center for Cancer, Tianjin Medical University Cancer Institute and Hospital, Tianjin 300060, China; 4 Key Laboratory of Cancer Prevention and Therapy, Tianjin 300060, China; 5 Tianjin’s Clinical Research Center for Cancer, Tianjin 300060, China; 6 Fujian Provincial Key Laboratory for Advanced Micro-Nano Photonics Technology and Devices, Research Center for Photonics Technology, Quanzhou Normal University, Fujian 362046, China

**Keywords:** MQD@ZIF-8, chemotherapy, photothermal therapy, photodynamic therapy, anti-tumor

## Abstract

It is critical to construct stimuli-responsive multifunctional nanoparticles for the drug delivery system for cancer treatment. Zeolitic imidazolate framework-8 (ZIF-8) has a large specific surface area and decomposes quickly under acidic conditions, which presents an excellent potential in pH-sensitive drug carriers. However, the mere chemotherapeutic drug loaded into ZIF-8 is a monotherapy and may restrict the therapeutic efficacy of malignancies. In this work, an effective nanoparticle-based delivery platform is established to simultaneously encapsulate doxorubicin (DOX) and MXene quantum dot (MQD) in ZIF-8 nanoparticles (MQD@ZIF-8/DOX). Under near-infrared (NIR) laser (808 nm) and UV light (365 nm) irradiation, MQD@ZIF-8 demonstrates a high photothermal conversion efficiency and reactive oxygen species (ROS) production, which shows excellent photothermal therapy and photodynamic therapy effects. Furthermore, the release of DOX-loaded into MQD@ZIF-8 nanoparticles is significantly increased under NIR laser irradiation and at pH 5.6, indicating that acidic conditions and NIR laser irradiation can be effectively combined to stimulate the drug release. The cellular experiments show that MQD@ZIF-8/DOX has an obvious killing effect on HeLa cells and achieves the combined anti-tumor effect of chemotherapy and phototherapy.

## 1. Introduction

A recent report suggests that malignant tumors remain a serious threat to human health and life [1]. Hence, the development of effective cancer treatments is of great importance [2,3]. Chemotherapy is one of the main treatment methods for malignant tumors. Unfortunately, chemotherapy often has adverse effects on healthy tissue cells, and the chemotherapeutic drugs alone are insufficient to destroy cancer cells. It is necessary to develop an intelligent drug delivery platform to treat malignancies more effectively [4]. To better treat tumors, several approaches have been investigated. Combination therapy combines two or more treatment forms, which offers a promising path to treating cancer successfully [5]. Among the many combination therapies, phototherapy is of great interest due to its minimal invasion, slight drug resistance, and low toxicity [6,7,8,9]. Phototherapy mainly relies on laser energy to produce adverse factors to kill cancer cells and realizes high spatiotemporal accuracy [10].

Phototherapy includes photothermal therapy (PTT) and photodynamic therapy (PDT). PTT converts light energy into heat by using the photothermic agent (PTA), resulting in local hyperthermia of the surrounding environment to kill the tumor. Compared with traditional cancer treatment methods, PTT has the advantages of non-invasiveness, low toxicity, and high anti-tumor efficiency [11]. PTAs, such as Au [12,13,14], Ag [15,16,17], and Pd [18,19,20] metal nanoparticles, play an important role in PTT. However, these metal nanomaterials are limited in the biological field due to their high cost and high toxicity; it is crucial to select a suitable PTA. PDT is also an important method of phototherapy because of its high precision, good applicability, and repeatable treatment [21]. When the photosensitizer (PS) is irradiated by laser, the energy is transferred to the surrounding oxygen molecules to generate toxic reactive oxygen species (ROS), which leads to cancer cell death [22]. However, two independent agents (PTA and PS) are required to activate PTT and PDT, respectively, resulting in a complex experimental process [23]. Furthermore, materials with several components have complicated interactions and degradation tendencies in biological systems, which might result in unidentified toxicity in the body [24]. Therefore, it is essential to develop a novel nanomaterial with both PTT and PDT properties [25]. MXene quantum dot (MQD) has attracted much attention due to the localized surface plasma resonance (LSPR) effect [26] and good biocompatibility. According to previous research, MQD is not only a photosensitizer but also a photothermal agent, so it endows a substance to have the PDT/PTT effect at the same time [27]. However, MQD has been discovered to be prone to agglomeration in solution, which can reduce its PDT and PTT effects and lead to poor transportation to the target tumor site for action [28].

A suitable drug delivery system must be chosen to address these issues. Metal-organic frameworks (MOFs) are a new type of porous material with metal ions/clusters as nodes and organic ligands as linkers. Due to high specific surface area and adjustable pore structure, MOFs have great prospects for applications in energy [29,30,31,32,33], catalysis [34], separations [35], and other fields [36]. In recent years, the encapsulation of drug molecules by MOFs has opened up a new way to obtain intelligent nanocarriers [37,38]. Zeolitic imidazolate framework-8 (ZIF-8), a low toxicity MOF, consists of Zn ions (Zn^2+^) and 2-methylimidazole (2-MIM) [39,40]. As a result of tunable composition, easy modification, and good biocompatibility, ZIF-8 can efficiently carry drugs, fluorescent molecules, and nanoparticles [41]. In addition, ZIF-8 is stable under physiological conditions, but is prone to decompose in a micro acidic environment such as the tumor microenvironment. The property allows ZIF-8 to be used as a pH-sensitive drug delivery carrier. 

**Scheme 1:**
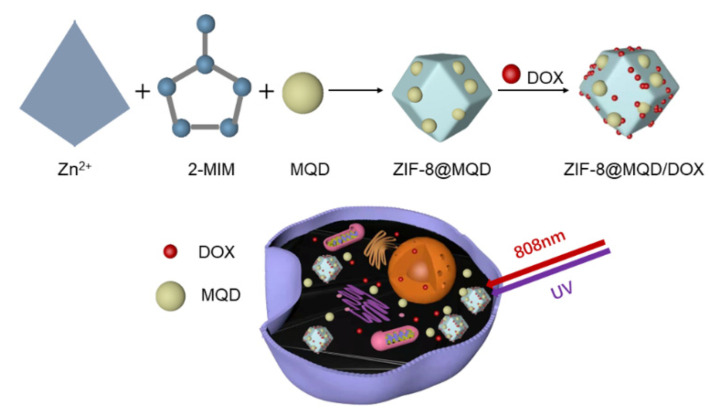
Schematic illustration of the MQD@ZIF-8/DOX fabrication process and the combined treatment for tumors stimulated by phototherapy and chemotherapy.

In this work, we designed a multifunctional MQD@ZIF-8 drug delivery platform. As illustrated in Scheme 1, Zn^2+^ was immobilized on MQD as nucleation nodes. MQD@ZIF-8 composites were in situ synthesized through the rapid reaction between Zn^2+^ and 2-MIM molecules. The chemotherapeutic drug doxorubicin (DOX) was encapsulated in MQD@ZIF-8 nanoparticles with loading capability as high as 89.5%. The structure of ZIF-8 can be collapsed in an acidic environment due to the pH-responsive property, and the releasing rate of MQD and DOX was therefore accelerated. MQD served as PTA and PS in this system to realize combined PDT/PTT therapy, which rapidly converted NIR light energy to ablative heat and produced ROS under UV laser irradiation to kill cancer cells. Moreover, DOX release could be markedly increased under the tumor environment and 808 nm laser irradiation, controlled by the pH and photothermal response. The MQD@ZIF-8/DOX nanocarrier can therefore achieve the effect of combined chemotherapy and phototherapy during tumor treatment [42].

## 2. Materials and Methods

### 2.1. Materials

Zinc nitrate hexahydrate (Zn(NO_3_)_2_⋅6H_2_O) and 2-MIM were supplied by Shanghai Aladdin Bio-Chem Technology Co. (Shanghai, China). MQD solution (2 mg/mL) was obtained from Beijing Beike New Material Technology Co. (Beijing, China). 2′,7′-dichlorofluorescein diacetate (DCFH-DA), Dulbecco’s minimum essential medium (DMEM), DOX, fluorescein isothiocyanate (FITC), and phosphate-buffered saline (PBS) were supplied from Solarbio Science and Technology Co., Ltd. (Beijing, China). 1,3-diphenylisobenzofuran (DPBF) were purchased from Shanghai McKellin Biochemical Technology Co., Ltd. (Shanghai, China). HeLa cells were obtained from Tianjin Cancer Hospital.

### 2.2. Synthesis of MQD@ZIF-8

One milliliter of the MQD solution (2 mg/mL) was dispersed in 10 mL of Zn(NO_3_)_2_·6H_2_O (0.1 mol/L) in methanol solution. Then, 10 mL of 2-MIM (0.8 mol/L) in methanol was added to the Zn(NO_3_)_2_·6H_2_O/MQD solution, followed by stirring for 1 h at 25 °C. The mixture was obtained by centrifugal separation and then washed 3 times with methanol.

### 2.3. Materials Characterization

The crystal structure was measured by X-ray diffraction (XRD, Bruker D8 Discover, Germany). The morphology was observed by scanning electron microscopy (SEM, Hitachi, Japan) and transmission electron microscopy (TEM, JEM2100F, JEOL, Japan). The charge was measured on Zeta potential analysis of macroscopic solid surface (SURPASS 3, Anton Paar GmbH, China). The surface area was assessed by Brunauer–Emmett–Teller (BET, V-Sorb 2800P, China). The functional group was recorded by Fourier transform infrared (FT-IR, TENSOR 27, Bruker, Germany). The UV-Vis spectra were performed by a UV-Vis spectrophotometer (UV-6100, Mapada, China).

### 2.4. DOX Loading and Release

To test the loading of the anticancer medication, 10 mg MQD@ZIF-8 was dispersed in the PBS solution of DOX (1 mg/mL) under the dark condition; the mixture was put in a reciprocating shaker at a speed of 150 rpm for 24 h. Then, the solid and liquid were then centrifuged for 5 min at 8000 rpm. MQD@ZIF-8/DOX was obtained by washing the solid 3 times with PBS and then freeze-drying. DOX content in the supernatant was determined by UV-Vis spectrophotometer, which was the mass of unencapsulated DOX. The loading efficiency (*LE*) of DOX was calculated according to the following equation.

$$LE=\frac{M_{x}-M_{y}}{M_{x}}\times 100\%$$

where $M_{x}$ is the total mass of DOX, and $M_{y}$ is the mass of unencapsulated DOX.

To demonstrate the drug release of the sample under pH and NIR stimulation, the as-prepared MQD@ZIF-8/DOX (10 mg) was dispersed in 5 mL PBS of different pH values (pH 7.4 and 5.6), placing them in the reciprocating shaker. The supernatant was collected at regular intervals by centrifugation, and the release of DOX was recorded by UV-Vis spectrophotometer. For photothermal-triggered drug release, the MQD@ZIF-8/DOX solution was irradiated under 808 nm NIR laser (2.0 W/cm^2^) for 5 min and then tested by UV-Vis spectrophotometer. The experiment was repeated by replenishing fresh equal amounts of PBS.

$$\mathrm{DOX}\;\mathrm{release}\left(\%\right)=\overset{n}{\underset{i=1}{\sum }}M_{i}/M_{0}\times 100\%$$

where $M_{i}$ is the amount of DOX released from MQD@ZIF-8/DOX at time i and $M_{0}$ is the total amount of loaded drug in MQD@ZIF-8.

### 2.5. Photothermal Effect of MQD@ZIF-8

The photothermal conversion ability of MQD@ZIF-8 was investigated. Firstly, the different concentrations (0.125, 0.25, and 0.5 mg/mL) of MQD@ZIF-8 in deionized water were irradiated by 808 nm NIR laser at 2.0 W/cm^2^ for 5 min. Next, the MQD@ZIF-8 solutions (0.5 mg/mL) were irradiated with different laser power densities (1.0, 1.5, and 2.0 W/cm^2^) to evaluate the effect of different laser power densities on photothermal performance. Finally, under laser irradiation at 808 nm (2 W/cm^2^), the temperature of MQD@ZIF-8 (0.5 mg/mL) was increased until it stabilized and then cooled to room temperature, 5 cycles were recorded to verify the photostability. Temperature variations were tracked in real-time using an FLIR (FLIR E95, Estonia) infrared thermal camera.

### 2.6. Photodynamic Effect of MQD@ZIF-8

Detection of singlet oxygen (^1^O_2_): In general, DPBF was selected as the ^1^O_2_ trapping agent. Under the dark condition, N,N-Dimethylformamide (DMF) solution of DPBF (1 mL, 200 μg/mL) was added to 9 mL of MQD@ZIF-8 in DMF solution (0.55 mg/mL). The mixture was irradiated under UV light for 20 min, and the absorbance of DPBF was detected every 5 min by a UV-Vis spectrophotometer. In addition, the absorbance of free DPBF under UV light was also measured under the same conditions.

*In vitro* ^1^O_2_ detection: HeLa cells were seeded into 96-well plates with a density of 5 × 10^4^ cells per well and cultured in DMEM medium for 24 h at 37 °C and 5% CO_2_. Next, the medium was removed, and the MQD@ZIF-8 sample (0.25 mg/mL) was co-cultured with HeLa cells for 2 h. After that, the cells were treated with DCFH-DA for 20 min and were exposed to UV light for 10 min. When the ROS was produced in cells, DCFH-DA can be oxidized to form fluorescent DCF. 4′,6-diamidino-2-phenylindole (DAPI, 100 nM) was used to label the nucleus. Confocal laser scanning microscopy (CLSM, TCSSP5II, Leica, Ernst-Leitz-Strasse, Wetzlar, Germany) was used to capture fluorescence pictures. The ^1^O_2_ generation capacity was measured by fluorescence intensity at 525 nm and 488 nm.

### 2.7. Cell Culture and Cell Cytotoxicity

Cell culture: HeLa cells were cultured into 96-well plates with a density of 5 × 10^4^ cells per well for 24 h. The medium was then removed, and the FITC-labeled MQD@ZIF-8 (0.25 mg/mL) was introduced to the medium to co-culture with HeLa cells for 4, 8, and 12 h. The cells were cleaned 3 times with PBS and stained with 4% paraformaldehyde for 20 min. The nucleus was labeled by DAPI, and the fluorescence pictures were obtained by CLSM.

Cell Cytotoxicity: The cytotoxicity was determined by the cell counting kit-8 (CCK-8, Abbkine Scientific Co., Ltd., Beijing, China). In a 96-well plate, HeLa cells were plated at a density of 1 × 10^4^ cells per well for 24 h. Firstly, the sterilized samples with concentrations of 50 μg/mL, 25 μg/mL, and 0 μg/mL were dispersed in the fresh medium. For the non-laser group, the medium of HeLa cells was removed, and the same amount of medium containing materials was co-cultured with cells for 24 h. For the laser irradiation group, the materials and HeLa cells were first co-cultured for 12 h, and then irradiated with 808 nm laser (2 W/cm^2^, 10 min), UV (10 min), 808 nm laser (2 W/cm^2^, 10 min) + UV (10 min), respectively, followed with another 12 h culture. After the above co-culturing for 24 h, the PBS buffer was used to wash the HeLa cells, and each well was filled with a combination of 10 μL of CCK-8 solution. After 3 h culture in an incubator, the OD values of the HeLa cells at 450 nm and the 96-well plate at 630 nm were detected by an enzyme labeling instrument, and then the OD value of 450–630 nm was used to evaluate the changes in cell proliferation.

### 2.8. Statistical Analysis

The results were reported as mean ± SD and were assessed statistically using the one-way analysis of variance (ANOVA). Statistical significance was accepted at *p* < 0.05.

## 3. Results and Discussions

### 3.1. Samples Characterization

**Figure 1:**
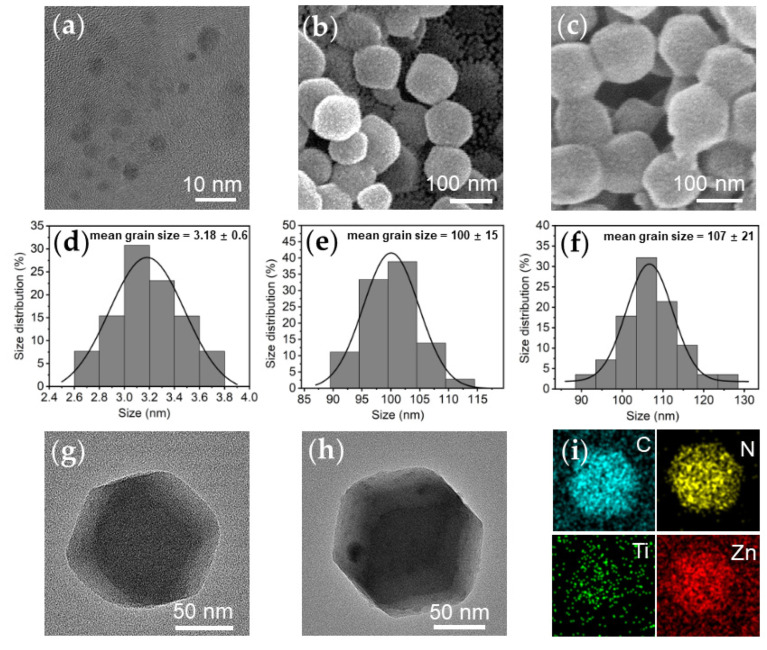
(**a**) TEM image of MQD; (**b**,**c**) SEM images of ZIF-8 and MQD@ZIF-8 samples; (**d**–**f**) size distributions of MQD, ZIF-8, and MQD@ZIF-8 samples. (**g**,**h**) TEM images of ZIF-8 and MQD@ZIF-8 samples. (**i**) Element mapping of MQD@ZIF-8 sample.

TEM and SEM were used to observe the morphologies of samples. MQD exhibited a good dispersion with a diameter of about 2~10 nm (Figure 1a) [43]. ZIF-8 presented as a rhombic dodecahedral crystal with uniform dispersion and a size of about 100 nm (Figure 1b,g). After the addition of MQD, the morphology of ZIF-8 did not change much; which was still a rhomboidal dodecahedron, but with a slight increase in size (Figure 1c,h). The size distributions of MQD, ZIF-8, and MQD@ZIF-8 were approximately 3.18 ± 0.6, 100 ± 15, and 107 ± 21 nm, respectively (Figure 1d–f). The TEM image shows apparent black nanodots in MQD@ZIF-8, which cannot be observed in pure ZIF-8. The black nanodots were very consistent with pure MQD (Figure 1g,h), proving that MQD was successfully loaded on the surface of ZIF-8 [44]. High magnification elemental mapping was also performed at a selected area, which further demonstrated the homogeneous element distribution of the obtained MQD@ZIF-8 composite (Figure 1i).

**Figure 2:**
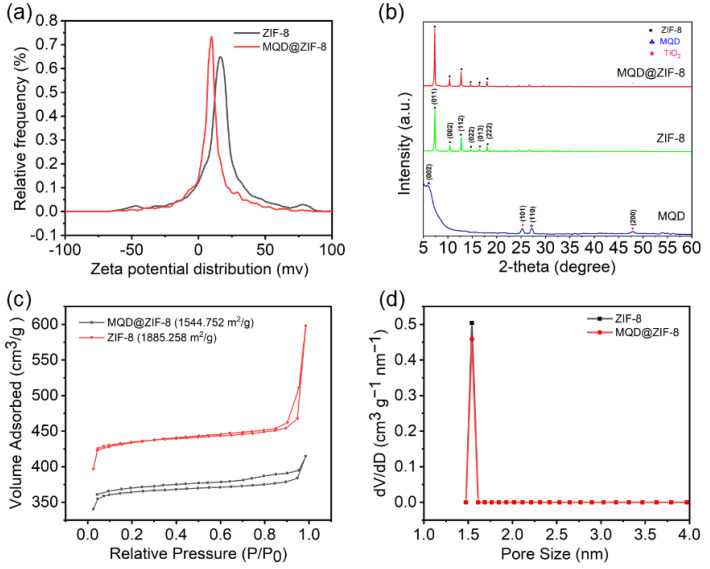
(**a**) Zeta potential spectra of ZIF-8 and MQD@ZIF-8 samples; (**b**) XRD patterns of ZIF-8, MQD, and MQD@ZIF-8. (**c**,**d**) N_2_ adsorption–desorption isotherms and pore size distribution curves of ZIF-8 and MQD@ZIF-8 samples.

After that, the zeta potentials of nanomaterials were tested. Zeta potentials of ZIF-8 and MQD@ZIF-8 samples were 17.7 and 9.5 mV, respectively (Figure 2a). The free carboxyl group of MQD had a significant negative charge, which caused the charge reversal, and the change of zeta potential proved the successful loading of MQD [45]. Figure 2b shows the XRD pattern of the samples; the coherent diffractions of the (011), (002), and (112) planes were responsible for characteristic peaks of ZIF-8 at 2θ = 7.28, 10.33, and 12.69° [28]. The (002) and (110) peaks of MQD and the (101) and (200) peaks of the titanium dioxide (TiO_2_) (JCPDS Card No. 71-1168) can be presented in the curve of the MQD sample, which indicated that a part of MQD was oxidized to TiO_2_ [46]. Moreover, the peaks of MQD@ZIF-8 and ZIF-8 were consistent, showing that the loading of MQD did not influence the crystal structure of ZIF-8. The nitrogen adsorption–desorption isotherms of ZIF-8 and MQD@ZIF-8 nanoparticles showed the type I isotherms, revealing a microporous structure. In addition, the specific surface areas of ZIF-8 and MQD@ZIF-8 were 1885.258 and 1544.752 m^2^/g, respectively (Figure 2c), which indicated that some pores were filled with MQD. The maximum pore size distribution of MQD@ZIF-8 appeared at 1.6 nm, which was similar to that of the ZIF-8 sample (Figure 2d). It is proved that the loading of MQD did not affect the pore size of ZIF-8.

### 3.2. Loading and Releasing Profiles of DOX

**Figure 3:**
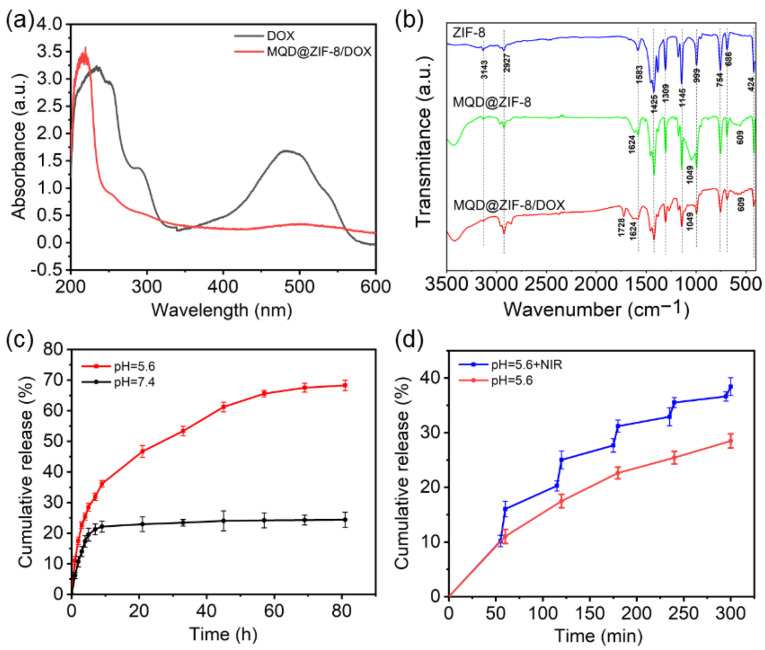
(**a**) UV–Vis absorption spectra of DOX and MQD@ZIF-8 /DOX; (**b**) FT-IR spectra of samples. (**c**,**d**) Release behaviors of MQD@ZIF-8/DOX under varied pH conditions and NIR irradiation.

DOX is a model chemotherapeutic drug. The loading of DOX was recorded by UV–Vis spectra (Figure 3a). DOX and MQD@ZIF-8/DOX exhibited absorbance at 480 nm and 500 nm, respectively. Here, the absorbance of MQD@ZIF-8/DOX was slightly red-shifted, possibly because of the interaction between DOX and ZIF-8, demonstrating the successful loading of DOX. In addition, the FT-IR spectra of DOX, MQD@ZIF-8, and MQD@ZIF-8/DOX samples were measured (Figure 3b). The bands at 3143 cm^−1^, 2927 cm^−1^, 1583 cm^−1^, and 1425 cm^−1^ were ascribed to the stretching vibrations of the C-H, and -NH- of the imidazole ring; the peaks at 1309, 1145, and 999 cm^−1^ were from the in-plane bending vibration of the imidazole ring, the peaks at 754 and 686 cm^−1^ were attributed to the out-of-plane bending vibration of the imidazole ring; the small peak near 424 cm^−1^ corresponded to the stretching vibration of Zn-N in ZIF-8. The obvious bands at 1624, 1049, and 609 cm^−1^ were assigned to the stretching vibrations of -OH, C-N, and Ti-C of the FT-IR spectra of MQD nanoparticles. Furthermore, the stretching vibration of C=O in the encapsulated DOX was found at 1728 cm^−1^, which corresponded with that of the free DOX. The characteristic peaks of DOX and MQD@ZIF-8 can be observed in the MQD@ZIF-8/DOX infrared spectrum, which indicated the successful synthesis of MQD@ZIF-8/DOX. Then, we tested the release behavior of DOX in PBS at pH 7.4 and 5.6; the release rate of DOX-loaded into MQD@ZIF-8 increased with the change of pH from 7.4 to 5.6. Nearly 70% of DOX was released from MQD@ZIF-8/DOX at pH 5.6 within 81 h, but just 24% was released at pH 7.4 (Figure 3c). Furthermore, DOX release was significantly increased under NIR laser irradiation (Figure 3d), proving that MQD@ZIF-8 can stimulate the release rate of DOX via adjusting pH values and NIR laser irradiation, which is favorable for combined chemical-photothermal treatment [47].

### 3.3. Analysis of Photothermal Properties of MQD@ZIF-8

**Figure 4:**
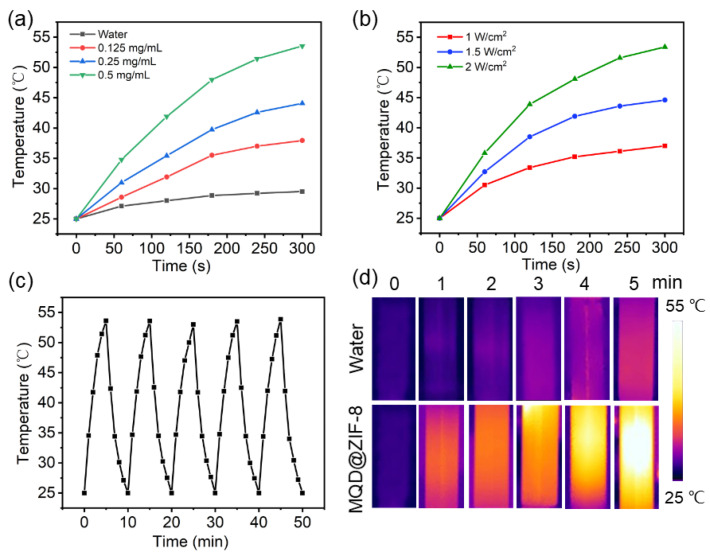
(**a**) Temperature increasing curves of water and different concentrations of MQD@ZIF-8 under irradiation of an 808 nm NIR laser at 2 W/cm^2^. (**b**) Temperature elevation of MQD@ZIF-8 dispersion (0.5 mg/mL) under varied laser power densities of an 808 nm NIR laser. (**c**) Photostability test of MQD@ZIF-8 solution under 808 nm NIR laser irradiation for 5 cycles (2 W/cm^2^, 5 min). (**d**) IR thermal images of water and MQD@ZIF-8 solution after irradiation for 5 min (808 nm, 2 W/cm^2^, 5 min).

Next, the photothermal properties of MQD@ZIF-8 were investigated. Under 808 nm NIR laser irradiation, we measured the temperature changes of MQD@ZIF-8 aqueous dispersions with different concentrations. After 5 min of laser irradiation (2 W/cm^2^), the temperature of the water group only increased by about 2 °C. However, the temperature of the MQD@ZIF-8 (0.5 mg/mL) solution could rise to 53.1 °C (Figure 4a), indicating that the temperature rose in the solution was due to the presence of MQD@ZIF-8. As shown in Figure 4b, when the NIR laser power increased, the temperature of the MQD@ZIF-8 aqueous solution also rose. In addition, the photothermal stability of the MQD@ZIF-8 solution was measured under 808 nm NIR laser irradiation, and the temperature profiles remained basically unchanged during the five-cycle heating and cooling process (Figure 4c) [48], which demonstrated that the MQD@ZIF-8 solution had excellent photothermal conversion efficiency. The IR thermal pictures were taken using the FLIR infrared camera (Figure 4d), which revealed that as the time passed, the temperature of the MQD@ZIF-8 solution increased. However, the temperature of the water group only rose a little, proving that MQD@ZIF-8 exhibited excellent photothermal effects [49].

### 3.4. Photodynamic Properties of MQD@ZIF-8

**Figure 5:**
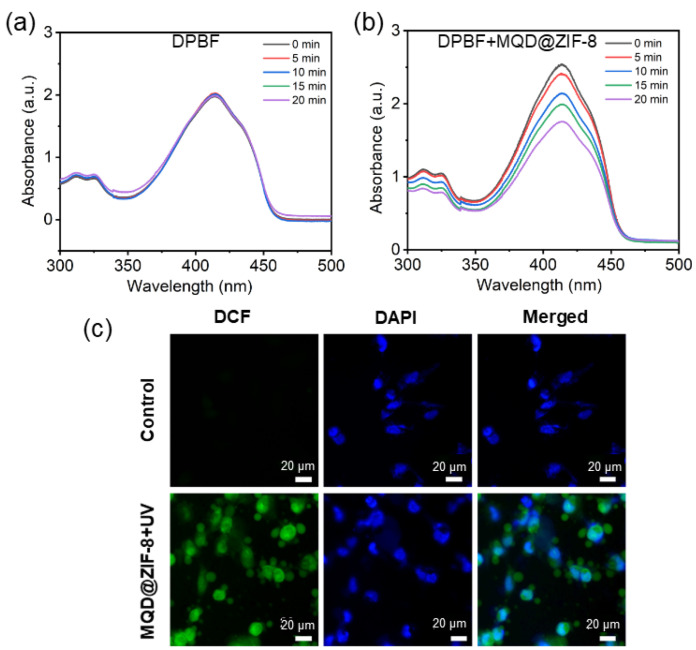
UV−Vis absorbance spectra of (**a**) DPBF and (**b**) DPBF with MQD@ZIF-8 under UV light irradiation; (**c**) confocal fluorescence images of ^1^O_2_ generation in HeLa cells.

To demonstrate whether the obtained MQD@ZIF-8 could generate ^1^O_2_ under UV light. Using the DPBF as a probe, DPBF can react with the ^1^O_2_ and then decomposed into 1,2-dibenzoylbenzene under laser irradiation, which resulted in the decrease of the absorption intensity at 410 nm. As shown in Figure 5a, the free DBPF had no noticeable change in UV-Vis absorbance spectra. However, the UV-Vis absorbance of DPBF incubated with MQD@ZIF-8 decreased with the laser irradiation time increasing from 5 min to 20 min (Figure 5b), which suggested that MQD@ZIF-8 can produce ^1^O_2_ due to the generation of TiO_2_. Furthermore, the ^1^O_2_ generation capability of MQD@ZIF-8 was observed in vitro. DCFH-DA was often used as an oxidation-sensitive probe to measure the ^1^O_2_ production in HeLa cells. When ^1^O_2_ was generated, DCFH-DA can be oxidized to DCF with fluorescent. The MQD@ZIF-8 group showed a large amount of intense green fluorescence under UV laser irradiated in confocal fluorescence images (Figure 5c), indicating the formation of toxic ROS in cells.

### 3.5. In Vitro Cellular Uptake

**Figure 6:**
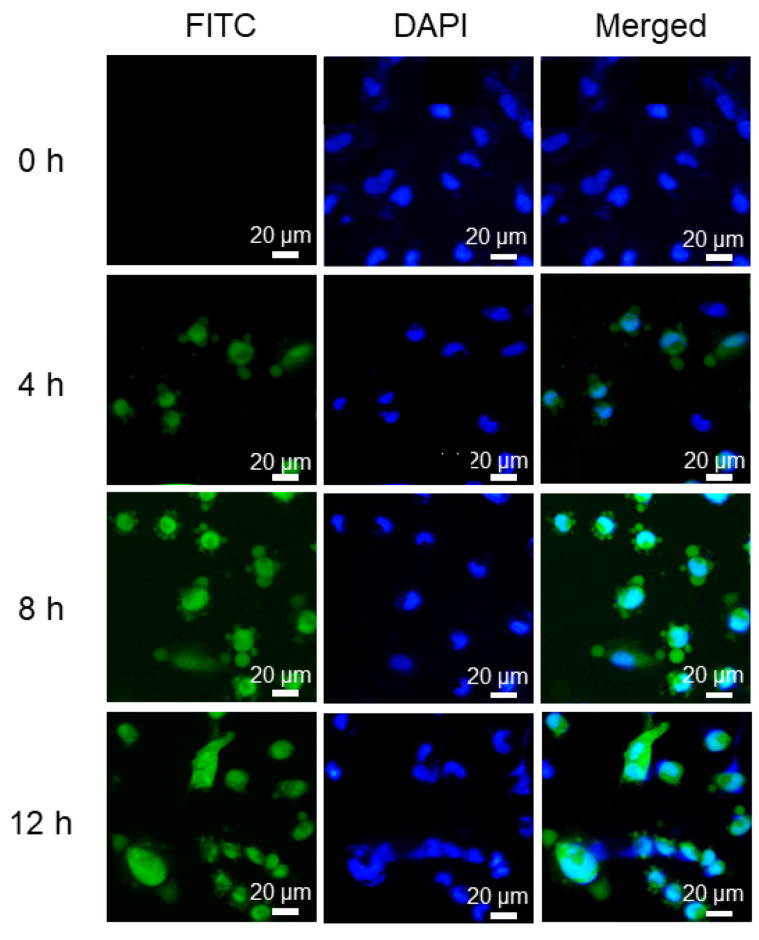
CLSM images of HeLa cells after being incubated with MQD@ZIF-8 for 0 h, 4 h, 8 h, and 12 h, respectively.

The uptake efficiency of MQD@ZIF-8 toward HeLa cells was evaluated via CLSM. HeLa cells were incubated with FITC labeled MQD@ZIF-8 for 4 h, 8 h, and 12 h, respectively. When incubated for 4 h, green fluorescence of FITC could be observed in HeLa cells (Figure 6). When the incubation time was prolonged to 12 h, MQD@ZIF-8 treated cells showed stronger green fluorescence, showing that MQD@ZIF-8 was internalized by the cells and had high uptake efficiency toward cancer cells.

### 3.6. Cell Cytotoxicity of MQD@ZIF-8

**Figure 7:**
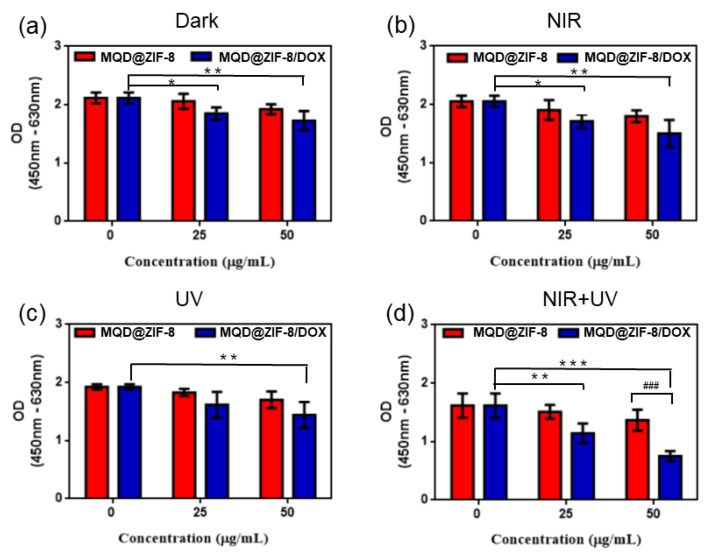
The OD value of HeLa cell with varied concentrations of MQD@ZIF-8 and MQD@ZIF-8/DOX samples for 24 h (**a**) under dark condition; (**b**) under 808 nm NIR irradiation; (**c**) under UV light irradiation; (**d**) under 808 nm NIR +UV light irradiation. Error bars represented standard deviation from the mean (*n* = 6). Asterisks indicated statistically significant differences (* *p* < 0.05, ** *p* < 0.01, ^###,^ *** *p* < 0.001).

In order to further study the biological application of MQD@ZIF-8 in vitro, the cytotoxicity of MQD@ZIF-8 was examined by using the CCK-8 assay. HeLa cells were co-cultured with diverse concentrations of MQD@ZIF-8 and MQD@ZIF-8/DOX for 24 h under dark conditions. According to the findings, MQD@ZIF-8 showed good biocompatibility and can be used as a drug delivery platform. However, the addition of DOX had a certain killing effect owing to the toxicity of the drug DOX itself (Figure 7a). Furthermore, we investigated the cytotoxicity from a combination of chemotherapy and photothermal therapy. The viability of HeLa cells incubated with MQD@ZIF-8 and MQD@ZIF-8/DOX significantly decreased under laser irradiation compared to that without irradiation, demonstrating that MQD@ZIF-8 can be used as PTA to effectively destroy the structure of cancer cells under the 808 NIR laser irradiation (Figure 7b). Moreover, to further research the PDT effect of MQD@ZIF-8 and MQD@ZIF-8/DOX in HeLa cells, the OD values of nanocomposites at various concentrations were tested under UV light irradiation. The sample had obvious cell death at the highest concentrations because of the production of ROS during the PDT process (Figure 7c). In addition, compared with the non-laser group, MQD@ZIF-8-treated cells with 808 nm laser and UV light irradiation had a significant difference. Furthermore, HeLa cells had substantially greater apoptotic than those treated with a single laser, demonstrating that combined therapy could significantly strengthen the therapeutic effect (Figure 7d).

## 4. Conclusions

In conclusion, a well-designed multifunctional MQD@ZIF-8 drug delivery system was created by simultaneously encapsulating MQD with phototherapeutic effects and the chemotherapeutic drug DOX in a ZIF-8 matrix. The MQD@ZIF-8 nanocarrier not only has a simple preparation method but can also efficiently connect pH with NIR dual responsive to stimulate drug release. In addition, through the cell experiment, it can be proved that MQD@ZIF-8 has superior biocompatibility, and MQD@ZIF-8 can generate heat and ROS around cells under 808 nm laser and UV irradiation to kill HeLa cells, respectively. Therefore, MQD@ZIF-8 is a nanoplatform with great potential for combination therapy of tumors.

## Data Availability

The data presented in this study are available on request from the corresponding author. The data are not publicly available due to privacy.
